# Resource Use and Costs of Dengue: Analysis of Data from Phase III Efficacy Studies of a Tetravalent Dengue Vaccine

**DOI:** 10.4269/ajtmh.16-0952

**Published:** 2017-10-16

**Authors:** Hanna El Fezzazi, Marie Branchu, Gabriel Carrasquilla, Punnee Pitisuttithum, Ana Paula Perroud, Carina Frago, Laurent Coudeville

**Affiliations:** 1 Dengue Vaccination Impact and Economics, Sanofi Pasteur, Lyon, France;; 2 Ecole des Hautes Etudes en Santé Publique (EHESP)—ESSEC Business School, Cergy, France;; 3 Fundación Santa Fe de Bogotá, Bogotá, Colombia;; 4 Vaccine Trial Centre, Faculty of Tropical Medicine, Mahidol University, Bangkok, Thailand;; 5 Clinical Research and Development, Sanofi Pasteur, Sâo Paulo, Brazil;; 6 Clinical Sciences, Sanofi Pasteur, Singapore, Singapore

## Abstract

A tetravalent dengue vaccine (CYD-TDV) has recently been approved in 12 countries in southeast Asia and Latin America for individuals aged 9–45 years or 9–60 years (age indication approvals vary by country) living in endemic areas. Data on utilization of medical and nonmedical resources as well as time lost from school and work were collected during the active phase of two phase III efficacy studies performed in 10 countries in the Asia-Pacific region and Latin America (NCT01373281; NCT01374516). We compared dengue-related resource utilization and costs among vaccinated and nonvaccinated participants. Country-specific unit costs were derived from available literature. There were 901 virologically confirmed dengue episodes among participants aged ≥ 9 years (*N* = 25,826): corresponding to 373 episodes in the CYD-TDV group (*N* = 17,230) and 528 episodes in the control group (*N* = 8,596). Fewer episodes in the CYD-TDV group resulted in hospitalization than in the control group (7.0% versus 13.3%; *P* = 0.002), but both had a similar average length of stay of 4 days. Overall, a two-thirds reduction in resource consumption and missed school/work days was observed in the CYD-TDV group relative to the control group. The estimated direct and indirect cost (2014 I$) associated with dengue episodes per participant in the CYD-TDV group was 73% lower than in the control group (I$6.72 versus I$25.08); representing a saving of I$I8.36 (95% confidence interval [CI]:17.05–19.78) per participant with vaccination. This is the first study providing information on dengue costs among vaccinated individuals and direct confirmation that vaccination has the potential to reduce dengue illness costs.

## INTRODUCTION

Dengue is an important mosquito-borne acute viral disease ubiquitous throughout the tropical and subtropical regions of the world. There are 128 countries where the disease is endemic, encompassing a population of over 3.9 billion people—about 50% of the global population—at potential risk of infection and ensuring disease.^1^ The incidence of dengue has continually increased over the past decades, with expansion of the geographical range of transmission to previously unaffected countries.^2–4^ Consequently, the global economic and disease burden is high, and can be substantial in countries where the disease is endemic.^5,6^ Recent estimates for 2013 suggest that there were 58.4 million symptomatic infections (95% uncertainty interval [UI]: 24–122 million) including 13,586 fatal cases (95% UI: 4,200–34,700) with associated costs of US$8.9 billion (95% UI: 3.7–19.7 billion).^7^ In the Americas, dengue illness was estimated to cost from a societal perspective US$2.1 billion (2010 US$) annually between 2000 and 2007.^5^ Similarly, in southeast Asia, the economic burden of dengue was estimated at US$950 million (2010 US$) annually in 12 countries between 2001 and 2010.^6^ The cost per capita can be as high as US$14.99 in some endemic countries.^5,6^

A recombinant yellow fever-17D–dengue virus, live, attenuated, tetravalent dengue vaccine (CYD-TDV; Dengvaxia^®^, Sanofi Pasteur, Lyon, France) has recently been approved in Mexico, Brazil, Philippines, El Salvador, Paraguay, Indonesia, Singapore, Guatemala, Peru, Thailand, Bolivia, and Costa Rica. These approvals were based in part on data obtained during two large-scale pivotal phase III studies (CYD14 and CYD15) undertaken in countries in Asia-Pacific and Latin America.^8,9^ The studies are still on going to better define the efficacy and safety of CYD-TDV over the longer term. Alongside these two pivotal clinical trials, data on resource use related to dengue management were prospectively collected to estimate the impact of vaccination on the cost burden attributed to the illness. Although a number of studies have suggested that a dengue vaccine has the potential to be cost-effective or even cost-saving,^10–15^ none have used prospectively collected resource use and health outcome data associated with vaccination.

The objective of our study was to compare breakthrough dengue disease–related resource utilization, direct, and indirect costs among participants receiving CYD-TDV with those receiving placebo during the 25 months of the active surveillance phase of two large-scale pivotal phase III studies.^8,9^

## METHODS

### Study design.

This economic analysis was conducted alongside two similarly designed randomized, placebo-controlled multicenter trials with CYD-TDV undertaken in five countries in the Asia-Pacific region (Indonesia, Malaysia, Philippines, Thailand, and Vietnam) and five countries in Latin America (Brazil, Colombia, Honduras, Mexico, and Puerto Rico) during the first 25 months of the active phase surveillance. Full details of the design, conduct, and main clinical findings of these trials have been reported elsewhere (NCT01373281; NCT01374516).^8,9^ The two trials were conducted in accordance with good clinical practice guidelines and the principles of the Declaration of Helsinki. Study protocols, amendments, consent, and assent forms were approved by each site’s independent ethics committee or institutional review board prior to study commencement. Parents or legal guardians provided informed consent before participation, and written assent was obtained from older children, in compliance with the regulations of each country.

The two studies recruited 31,126 healthy children aged 2–16 years (2–14 years in Asia-Pacific and 9–16 years in Latin American) and randomly assigned them in a 2:1 ratio to receive CYD-TDV (*N* = 20,762) or placebo control (*N* = 10,364). Participants were given three doses of CYD-TDV or placebo at 0, 6, and 12 months, and actively monitored over 25 months after the first study injection for acute febrile illness (temperature ≥ 38°C on ≥ 2 consecutive days), and those who presented with fever were screened for signs and symptoms of dengue. The suspected febrile episodes were classified as virologically confirmed dengue if any of the following tests on acute and convalescent blood samples were positive: dengue nonstructural protein (NS1) antigen enzyme-linked immunosorbent assay (Platelia^TM^, Bio-Rad Laboratories), and serotype-specific dengue reverse transcriptase polymerase chain reaction.

### Resource use and missed school days/workdays.

Data on resource utilization (medical and nonmedical resource use) as well as productivity losses for participants and parents/caregivers due to time lost from school and work were prospectively documented in Case Report Forms during each study visit for those with suspected dengue episodes during the first 25 months of the active phase surveillance. Data included information on where the participant consulted (at trial site or elsewhere), hospitalizations (at trial site or elsewhere and type of ward), medications, inpatient and outpatient tests, type of travel used to get to the health center and absenteeism (number of days missed from work or school by the participant or their caregivers).

### Resource unit costs.

Estimates of country-specific unit costs were derived from available literature for the individual countries (see Supplemental Appendices 1–10). All country-specific costs were expressed in 2014 US$ and included direct (i.e., related to resource utilization) and indirect costs (i.e., related to productivity losses). If country-specific cost estimates were only available for years earlier than 2014, these were updated by initially adjusting the estimates to 2014 local currency values accounting for the inflation rate of the country before converting to 2014 US$. If country-specific unit costs were not available, then unit costs were extrapolated from data available from other countries in the region. To assess average unit costs across the 10 countries included in this study (see Supplemental Appendix 11), country-specific unit costs were further converted to 2014 international dollars (I$), which adjust for differences in the relative price of the resources used across countries based on purchasing power parity at official exchange rates, and thereby allows cost information from different countries to be combined.^16^ Inflation rates, exchange rates, and purchasing power parity used for these calculations were based on International Monetary Fund statistics (see Supplemental Appendix 12 for details).^17^

A macrocosting method was used for assessing hospitalization costs,^18^ that is, the cost per hospitalization day used included fixed costs and personnel costs as well as examination and treatment costs. We considered public hospital for the Latin and Asian countries of the studies. Outpatient and ambulatory costs, incurred at the trial site or another health-care center, respectively, were calculated using a similar macro-costing method used for assessing hospitalization costs, and included consultation, examination, and treatment costs. The cost of travel undertaken with personal vehicles was estimated on a per kilometer basis assuming an average of 10 kilometers per reported trip. For travel by taxis or ambulance, the cost was calculated by adding the salary costs for the driver (assuming an average of 30 minute per ride and considering the average daily wage for the country). Lost productivity due to work days lost by the parent/caregiver was valued by using the average daily wage in each country.

### Statistical methods.

The cost per dengue episode was calculated by multiplying the resource quantities used with the assigned unit costs identified from the reference sources for the individual countries (Supplemental Appendices 1–10) summed across all items, divided by the total number of episodes. The cost per participant was calculated by multiplying the cost per dengue episode by the resource use incidence. For the purpose of our analysis, we focused mainly on virologically confirmed dengue in those aged ≥ 9 years corresponding to the age indication for this vaccine.^19^ Cost data for participants aged ≥ 9 years were also compared with that for those aged < 9 years.

Chi-squared tests were used to detect significant differences in frequency observed between groups with a 5% threshold for rejecting the independence between groups. A bilateral Student’s *t* test was used to compare the mean of two distributions at a 5% threshold for significant differences. Equality between variances (homoscedasticity) was assessed with the *F* test. Naive bootstrap was used for the calculation of 95% confidence intervals for percentage reductions with CYD-TDV (1,000 replications).

## RESULTS

### Participants with virologically confirmed dengue episodes.

There were 1,279 virologically confirmed dengue episodes (*N* = 31,126): 571 in the CYD-TDV group (*N* = 20,762) and 708 episodes in the control group (*N* = 10,364). The baseline characteristics of the virologically confirmed dengue cases are summarized in Table 1. There was no significant difference in participant characteristics between the two groups. Overall, there were 901 virologically confirmed dengue episodes among those aged ≥ 9 years (*N* = 25,826): 373 in the CYD-TDV group (*N* = 17,230) and 528 in the control group (*N* = 8,596).^20^

**Table 1:** Baseline characteristics of the participants all ages with virologically confirmed dengue (all countries combined)

	Participants	CYD-TDV group	Control group
All participants			
Participants (*n* [%])	1,259 [100]	564 [45]	695 [55]
Age (years [SD])	10.1 [3.5]	9.6 [3.7]	10.5 [3.3]
Sex (*n* [%])			
Male	635 [51]	271	364
Female	624 [49]	293	331
Participants aged ≥ 9 years			
Participants (*n* [%])	895 [100]	370 [41]	525 [59]
Age (years [SD])	12 [2]	11.9 [2]	12.1 [2]
Sex (*n* [%])			
Male	477 [53]	187	290
Female	418 [47]	183	235

SD = standard deviation.

### Resource utilization.

Medical and nonmedical resource use as well as missed school days/workdays for participants aged ≥ 9 years who had virologically confirmed dengue are summarized in Table 2. Data on resource use for all episodes and participants (all ages) combined are summarized in Supplemental Appendix 11. Approximately, twice as many episodes in the control group resulted in hospitalization than in the CYD-TDV group (13.3% versus 7.0%; *P* = 0.002), but the average length of stay was the same at 4.0 days in both groups. Dengue episodes in the control group resulted in significantly more missed school days (2.2 days versus 1.8 days; *P* = 0.004) and there was also a trend toward more work days lost by parents/caregivers in this group (Table 2).

**Table 2:** Medical and nonmedical resource use and missed school days/workdays attributed to virologically confirmed dengue episodes (participants aged ≥ 9 years, all countries combined)

	All groups (average)	CYD-TDV group (average)	Control group (average)	*P* value *t* test
Hospitalized	96 (10.7%)	26 (7%)	70 (13.3%)	0.002
Length of stay (hospitalization days)	4.0	4.0	4.0	0.98
Consultations (*N*)	3.0	3.3	2.8	0.16
Workdays lost	2.2	2.0	2.2	0.72
School days missed	4.9	4.4	5.1	0.44
Nonhospitalized	805 (89.3%)	347 (93%)	458 (86.7%)	
Consultations (*N*)	2.4	2.3	2.4	0.09
Workdays lost	0.3	0.3	0.3	0.58
School days missed	1.7	1.6	1.8	0.10
All episodes*	901	373	528	
Length of stay (hospitalization days)	0.4	0.3	0.5	0.007
Consultations (*N*)	2.5	2.4	2.5	0.15
Workdays lost	0.5	0.4	0.6	0.07
School days missed	2.0	1.8	2.2	0.004

* Weighted average.

Resource consumption and missed school/work days attributed to virologically confirmed dengue as a percentage of total number of participants in each study group and overall is summarized in Table 3—there was at least a two-thirds reduction in resource consumption and missed school/work days in the CYD-TDV group relative to the control group. Similar reductions in the levels of resource consumption and missed school/work days were observed by country (see Supplemental Appendices 1–10).

**Table 3:** Resource consumption and missed school/work days attributed to virologically confirmed dengue calculated per participant (participants aged ≥ 9 years, all countries combined)

	All groups (*N* = 25,826)	CYD-TDV group (*N* = 17,230)	Control group (*N* = 8,596)	% Change with CYD-TDV (central value [95% CI])
All countries combined				
Number of dengue cases	901	373	528	
Resource use incidence (%)*	3.49	2.16	6.14	
Length of stay		0.0061	0.0328	−81 [−91 to −72]
Consultations		0.0521	0.1532	−66 [−71 to −61]
Workdays lost		0.0092	0.0365	−75 [−84 to −66]
School day missed		0.0379	0.1356	−72 [−78 to −66]

CI = confidence interval.

Data shown as a percentage of total number of participants in each study group and overall.

Overall, dengue episodes in those aged ≥ 9 years generally consumed fewer resources and resulted in fewer missed school/work days than in those aged < 9 years (Table 4) (see Supplemental Appendix 13 for resource use among those aged < 9 years).

**Table 4:** Summary of resource use comparison between participants with virologically confirmed dengue episodes (participants aged ≥ 9 years vs. < 9 years, all countries combined)

	All groups (average)	≥ 9 years (average)	< 9 years (average)	*P* value (*t* test)
Resource consumption				
Hospitalized	158 (12.4%)	96 (10.7%)	62 (16.4%)	
Length of stay (hospitalization days)	4.1	4.0	4.3	0.003
Consultations (*N*)	3.2	3.0	3.5	0.0006
Workdays lost	2.1	2.2	1.9	0.0036
School days missed	4.9	4.9	4.9	0.078
Nonhospitalized	1,121 (87.6%)	805 (89.3%)	316 (83.6%)	
Consultations (*N*)	2.4	2.4	2.4	
Workdays lost	0.4	0.3	0.6	
School days missed	1.7	1.7	1.7	
All episodes*	1,279	901	378	
Length of stay(hospitalization days)	0.5	0.4	0.7	
Consultations (*N*)	2.5	2.5	2.6	
Workdays lost	0.6	0.5	0.8	
School days missed	2.1	2.0	2.3	

* Weighted average.

### Cost analysis.

The estimated average costs associated with virologically confirmed dengue episodes in those aged ≥ 9 years are summarized in Table 5. The estimated cost per dengue episode was higher in the control group than the CYD-TDV group (*P* = 0.002). The cost of work and school-related absenteeism were also higher in the control group (*P* = 0.004). There were no significant differences in consultation costs (*P* = 0.085) or travel-associated costs (*P* = 0.42) between the two groups. Country-specific estimated costs associated with hospitalized and nonhospitalized dengue episodes are summarized in Supplemental Appendices 1–10.

**Table 5:** Costs associated with virologically-confirmed dengue episodes (participants aged ≥ 9 years, all countries combined)

	All groups (average)	CYD-TDV group (average)	Control group (average)	*P* value *t* test
Hospitalized episodes (all countries combined)				
Hospitalization costs	1,115.52	1,117.52	1,114.78	
Consultation costs	211.17	232.54	203.23	
Outpatient consultations	167.96	190.81	159.47	
Ambulatory consultations	43.21	41.73	43.76	
Travel costs	5.47	5.41	5.49	
Absence costs	221.60	201.54	229.05	
Total cost per dengue episode	1,553.76	1,557.01	1,552.55	
Nonhospitalized episodes (all countries combined)				
Consultation costs	159.63	154.62	163.43	
Outpatient consultations	140.13	137.33	142.24	
Ambulatory consultations	19.50	17.29	21.18	
Travel costs	4.06	3.97	4.12	
Absence costs	62.95	58.32	66.45	
Total cost per dengue episode	226.64	216.91	234.00	
All episodes*				
Hospitalization costs	118.86	77.90	147.79	0.007
Consultation costs	165.12	160.05	168.70	0.085
Outpatient consultations	143.09	141.06	144.53	
Ambulatory consultations	22.03	18.99	24.18	
Travel costs	4.21	4.07	4.30	0.42
Absence costs	79.85	68.31	88.01	0.004
Total cost per dengue episode	368.04	310.32	408.81	0.002

Data shown in 2014 I$.

* Weighted average.

The estimated costs of virologically confirmed dengue per participant aged ≥ 9 years are summarized in Table 6. The estimated total costs per participant in the CYD-TDV group were 73% lower than in the control group (I$6.72 versus I$25.08); representing a saving of I$I8.36 (95% CI: 17.05–19.78) per participant with vaccination. Country-specific estimated costs per participant are also summarized in Supplemental Appendices 1–10.

**Table 6:** Costs associated with virologically confirmed dengue episodes per participant (participants aged ≥ 9 years, all countries combined)

	All participants (average)	CYD-TDV group (average)	Control group (average)	% Change with CYD-TDV (central value [95% CI])
All countries combined				
Hospitalization costs	4.14	1.69	9.07	−81% [−91 to −73]
Consultation costs	5.76	3.46	10.35	−67% [−72 to −62]
Outpatient consultations	4.99	3.05	8.87	−66% [−71to −61]
Ambulatory consultations	0.77	0.41	1.48	−72% [−82 to −64]
Travel costs	0.15	0.09	0.26	−67% [−73 to −61]
Absence costs	2.78	1.48	5.40	−73% [−79 to −67]
Total cost of dengue per participant	12.83	6.72	25.08	−73% [−79 to −68]

CI = confidence interval. Data shown as cost per participant in 2014 I$.

## DISCUSSION

This is the first study to show directly that CYD-TDV has the potential to reduce dengue illness costs among participants aged ≥ 9 years, the age group in which vaccine use has been approved. In this age group, we showed that breakthrough dengue episodes in the CYD-TDV group (versus control group) lead to fewer hospitalizations, consumed fewer resources, and resulted in fewer missed school/work days. The associated costs were therefore also lower per participant in the CYD-TDV groups than in the control group. This result is mainly driven by a lower proportion of hospitalized dengue episodes in the CYD-TDV group during the active phase surveillance. Overall, pooled vaccine efficacy rates against symptomatic virologically confirmed dengue during the first 25 months were 60.3% (95% CI: 55.7–64.5) for all participants and 65.6% (95% CI: 60.7–69.9) for those aged ≥ 9 years (Cox Regression).^20^ Our study confirms the results of previous modeling studies suggesting that a dengue vaccine could be of economic value in dengue-endemic setting.^11,12,21^

Resource use among participants hospitalized for dengue across both groups in our study, in terms of length of stay, number of consultations, workdays and school days missed, appear broadly consistent with those reported in another study undertaken in Thailand among hospitalized dengue patients aged 2–15 years^22^: length of stay (4.0 days in our study versus 5.5 days in the later); number of consultations (3.0 versus 3.6); workdays lost (2.2 versus 4.5 days); and school days missed (4.9 versus 5.9 days). A similar length of stay was reported for hospitalized dengue patients aged < 15 years in Brazil; mean 5 (standard deviation: 5.2) days, with a trend toward shorter stays among those hospitalized in the private sector.^23^ Several other studies have reported median/mean stays in the range 3–6 days among the general population hospitalized for dengue.^16,24–28^ Moreover, the mean length of hospital stay was shown to increase with severity of the disease.^23^

The number of school days missed in our study (4.9 days for hospitalized and 1.7 days for nonhospitalized) is also consistent with other studies that reported an average 3.8–6.9 and 2.0–5.2 days of school lost among hospitalized and ambulatory patients, respectively.^16,29^ Another study that did not differentiate between hospitalized and ambulatory patients reported an average of 1.9 days of schools days lost.^28^ Similarly, the number of work days lost associated with hospitalized participants in our study (2.2 days) is also consistent with other studies that reported an average 3.1–14.4 days.^16,29^ However, the average number of work days lost associated with nonhospitalized participants (0.3 days) in our study appears somewhat lower than that reported elsewhere (1.7–6.0 days of work lost).^16,29^ Two other studies that did not differentiate between hospitalized and ambulatory patients reported 4.2 and 5.3 days of work missed, respectively.^28,30^

The observed total costs per hospitalized dengue episode in the vaccine group (I$1,557) were similar to those in the control group (I$1,553), as were nonhospitalized dengue episodes (I$217 and I$234, respectively). Although there is substantial variability in the costs per dengue episode by country,^5,6^ our estimated costs are within the ranges for previous estimates for countries in Latin America and Asia. A study of dengue illness that included 46 countries in Latin America and the Caribbean (excluding North America) reported median cost (2010 US$) per hospitalized and ambulatory cases of US$1,213 (range: US$306–US$7,411) and US$451 (range: US$72–US$2,300), respectively.^5^ Other single-country studies in Latin America have also reported similar per dengue episode costs.^23,28,29,31–38^ Similarly, in southeast Asia, another multicountry study of dengue illness (12 countries) reported median cost (2010 US$) per hospitalized and ambulatory cases as US$210 (range: US$66–US$3,009) and US$63 (range: US$19–US$1,268), respectively.^6^ Other individual country studies in Asia have also reported similar cost per dengue episode costs.^6,22,33,34,39–45^

Our study has a number of limitations that should be considered when evaluating our results and their relative generalizability. A weakness of this study relates to the potential inclusion of protocol-driven costs as the trial involves activities/contacts with the study investigators/health staff that may not be otherwise available in routine care. The exclusion criteria may lead to the selection of participants in specific geographic locations and may not necessarily be representative of or generalizable to the “typical” patient population or other regions. In addition, our analysis was based on studies undertaken in countries with high dengue endemicity and as such, may not be representative of countries with low dengue endemicity where disease treatment/management and practice may differ substantially. A major limitation, however, is that the vaccination costs were not included in the cost per episode calculations. In addition, sensitivity analyses on unit costs were not undertaken. Nonetheless, the unit costs would apply to both the CYD-TDV and control groups, and as such would not affect the relative cost ratio between the two groups.

The major strength of our study is its prospective design and standardized collection of resource use information as part of two randomized clinical trials, which also facilitates the comparisons between countries. The inclusion of a control group provided an opportunity to directly estimate the impact of vaccination on dengue-related costs. We also included indirect costs related to travel and time lost from school and work for patients and parents/caregivers. Our results suggest that CYD-TDV has the potential to reduce dengue illness costs among participants aged ≥ 9 years, the age group in which vaccine use has been approved.

## Supplementary Material

Supplemental Appendix.
