# Supplementary material for: Resource Use and Costs of Dengue: Analysis of Data from Phase III Efficacy Studies of a Tetravalent Dengue Vaccine
Source: Am J Trop Med Hyg. 2017 Oct 16;97(6):1898–903. doi: 10.4269/ajtmh.16-0952 (PMC5805027; doi:10.4269/ajtmh.16-0952)
Supplement: Supplementary file 1 [file tpmd160952.SD1.pdf]

SUPPLEMENTAL MATERIAL

APPENDIX 1: INDONESIA

Supplemental Table 1

Unit costs and source data (2014 US$)

Cost type	Unit cost (US$)	Source
Indonesia		
Hospitalization cost bed per day public	56.09	Shepard et al.[7]/Average number of hospitalization days CYD14; adjusted 2014 US$
Outpatient consultation public	13.59	Shepard et al.[7]/Average number of consultation CYD14; adjusted 2014 US$
Ambulatory consultation public	13.59	Shepard et al.[7]/Average number of consultation days CYD14; adjusted 2014 US$
Daily wage	6.71	World Bank GNIPC[11]
Personal car (hypothesis travel 10 km)	0.92	WHO-CHOICE[13]
Motorcycle (hypothesis travel 10 km)	0.34	WHO-CHOICE[13]
Taxi (cost per travel, hypothesis 10 km in 1/2 hours)	1.26	= Cost per car for 10 km + 1/2 hour of daily wage (WHO-CHOICE[13]; World Bank GNIPC[11])
Public transportation (cost per travel)	0.13	Vietnam cost study Sanofi Pasteur 2012 (Luong et al., 2012), adjusted by 2014 I$ ratio (IMF[4])
Ambulance (cost per travel, hypothesis 10 km in 1/2 h)	1.26	= Cost per car for 10 km + 1/2 hours of daily wage (WHO-CHOICE[13]; World Bank GNIPC[11])

Supplemental Table 2

Medical and nonmedical resource use and missed school days/workdays attributed to virologically confirmed dengue episodes for all participants (2–14 years) in Indonesia

Indonesia	All episodes (average)	CYD-TDV group (average)	Control Group (average)	*P* value *t* test
Hospitalized	28 (33.3%)	8 (20.0%)	20 (45.5%)	
Length of stay (hospitalization days)	5.1	4.0	5.5	< 0.05
Consultations (*N*)	3.4	3.3	3.4	NS
Workdays lost	1.7	1.4	1.8	NS
School days missed	5.3	5.1	5.4	NS
Nonhospitalized	56 (66.7%)	32 (80.0%)	24 (54.5%)	
Consultations (*N*)	2.6	2.5	2.7	NS
Workdays lost	0.1	0.2	0.1	NS
School days missed	2.1	2.1	2.1	NS
All cases	84	40	44	
Length of stay (hospitalization days)	1.7	0.8	2.5	NS
Consultations (*N*)	2.9	2.7	3.0	NS
Workdays lost	0.6	0.4	0.9	NS
School days missed	3.2	2.7	3.6	NS

NS = not significant.

Supplemental Table 3

Costs associated with virologically confirmed dengue episodes (hospitalized and nonhospitalized combined) for all participants (2–14 years) in Indonesia

	All participants (average)	CYD-TDV group (average)	Control group (average)	Reduction (%)
Indonesia				
Incidence (%)	2.61	1.77	4.26	
Hospitalization costs	2.01	0.62	4.91	87.31
Consultation costs	1.37	0.85	2.41	64.73
Outpatient consultations	1.31	0.83	2.27	63.30
Ambulatory consultations	0.05	0.02	0.14	88.60
Absence costs	0.01	0.01	0.03	76.38
Travel costs	0.67	0.37	1.27	70.91
Total cost of dengue per participant	4.06	1.85	8.62	78.54

Data shown as cost per participant using 2014 US$ unless otherwise stated.

APPENDIX 2: MALAYSIA

Supplemental Table 4

Unit costs and source data (2014 US$)

Cost type	Unit cost (US$)	Source
Malaysia		
Hospitalization cost bed per day public	277.77	Shepard et al.[8]; adjusted 2014 US$
Outpatient consultation public	53.18	Shepard et al.[8]; adjusted 2014 US$
Ambulatory consultation public)	53.18	Shepard et al.[8]; adjusted 2014 US$
Daily wage	24.55	World Bank GNIPC[11]
Personal car (hypothesis travel 10 km)	1.23	WHO-CHOICE[13]
Motorcycle (hypothesis travel 10 km)	0.43	WHO-CHOICE[13]
Taxi (cost per travel, hypothesis 10 km in 1/2 hour)	2.46	= Cost per car for 10 km + 1/2 hours of daily wage (WHO-CHOICE[13]; World Bank GNIPC[11])
Public transportation (cost per travel)	0.68	Vietnam cost study Sanofi Pasteur 2012 (Luong et al., 2012), adjusted by 2014 I$ ratio (IMF[4])
Ambulance (cost per travel, hypothesis 10 km in 1/2 hour)	2.46	= Cost per car for 10 km + 1/2 hour of daily wage (WHO-CHOICE[13]; World Bank GNIPC[11])

Supplemental Table 5

Medical and nonmedical resource use and missed school days/workdays attributed to virologically confirmed dengue episodes for all participants (2–14 years) in Malaysia

Malaysia	All episodes (average)	CYD-TDV group (average)	Control group (average)	*P* value *t* test
Hospitalized	11 (36.7%)	3 (11.1%)	8 (38.1%)	
Length of stay (hospitalization days)	4.5	4.3	4.6	NS
Consultations (*N*)	2.7	1.7	3.1	NS
Workdays lost	1.4	0.7	1.6	NS
School days missed	5.2	6.0	4.9	NS
Nonhospitalized	19 (63.3%)	6 (88.9%)	13 (61.9%)	
Consultations (*N*)	2.2	2.1	2.2	NS
Workdays lost	0.3	0.2	0.3	NS
School days missed	1.4	1.0	1.5	NS
All cases	30	9	21	
Length of stay (hospitalization days)	1.7	1.4	1.8	NS
Consultations (*N*)	2.4	2.0	2.5	NS
Workdays lost	0.6	0.4	0.8	NS
School days missed	2.5	2.7	2.8	NS

NS = not significant.

Supplemental Table 6

Costs associated with virologically confirmed dengue episodes (hospitalized and nonhospitalized combined) for all participants (2–14 years) in Malaysia

	All participants (Average)	CYD-TDV group (Average)	Control group (Average)	Reduction (%)
Malaysia				
Incidence (%)	2.12	1.06	4.26	
Hospitalization costs	9.84	4.26	20.83	79.55
Consultation costs	3.20	1.25	7.00	82.09
Outpatient consultations	2.79	1.25	5.82	78.44
Ambulatory consultations	0.41	0.00	1.19	100.00
Absence costs	0.08	0.03	0.17	80.95
Travel costs	1.79	0.78	3.78	79.32
Total cost of dengue per participant	14.91	6.33	31.78	80.09

Data shown as cost per participant using 2014 US$ unless otherwise stated.

APPENDIX 3: PHILIPPINES

Supplemental Table 7

Unit costs and source data (2014 US$)

Cost type	Unit cost (US$)	Source
Philippines		
Hospitalization cost bed per day public	71.25	Edillo et al.[2]; adjusted 2014 US$
Outpatient consultation public	22.80	Edillo et al.[2]; adjusted 2014 US$
Outpatient consultation public (other than hospital settings)	22.80	Edillo et al.[2]; adjusted 2014 US$
Daily wage	6.69	World Bank GNIPC[11]
Personal car (hypothesis travel 10 km)	1.27	WHO-CHOICE[13]
Motorcycle (hypothesis travel 10 km)	0.44	WHO-CHOICE[13]
Taxi (cost per travel, hypothesis 10 km in 1/2 hour)	1.60	= Cost per car for 10 km + 1/2 hours of daily wage (WHO-CHOICE[13]; World Bank GNIPC[11])
Public transportation (cost per travel)	0.12	Vietnam cost study Sanofi Pasteur 2012 (Luong et al., 2012); adjusted by 2014 I$ ratio (IMF[4])
Ambulance (cost per travel, hypothesis 10 km in 1/2 hour)	1.60	= Cost per car for 10 km + 1/2 hours of daily wage (WHO-CHOICE[13]; World Bank GNIPC[11])

Supplemental Table 8

Medical and nonmedical resource use and missed school days/workdays attributed to virologically confirmed dengue episodes for all participants (2–14 years) in Philippines

Philippines	All episodes (average)	CYD-TDV group (average)	Control group (average)	*P* value *t* test
Hospitalized	26 (8.6%)	9 (6.2%)	17 (10.9%)	
Length of stay (hospitalization days)	4.1	3.2	4.5	< 0.01
Consultations (*N*)	3.7	3.9	3.5	NS
Workdays lost	1.0	0.4	1.4	NS
School days missed	5.6	4.3	6.2	NS
Nonhospitalized	275 (91.4%)	136 (93.8%)	139 (89.1%)	
Consultations (*N*)	2.3	2.3	2.3	NS
Workdays lost	0.3	0.4	0.3	NS
School days missed	1.9	1.5	2.2	< 0.05
All cases	301	145	156	
Length of stay (hospitalization days)	0.4	0.2	0.5	< 0.05
Consultations (*N*)	2.4	2.4	2.4	NS
Workdays lost	0.4	0.4	0.4	NS
School days missed	2.2	1.7	2.6	<0.01

NS = not significant.

Supplemental Table 9

Costs associated with virologically confirmed dengue episodes (hospitalized and nonhospitalized combined) for all participants (2–14 years) in Philippines

	All participants (average)	CYD-TDV group (average)	Control group (average)	Reduction (%)
Philippines				
Incidence (%)	6.22	3.63	11.51	
Hospitalization costs	1.68	0.52	4.47	88.44
Consultation costs	3.91	2.23	7.38	69.82
Outpatient consultations	3.39	1.95	6.34	69.17
Ambulatory consultations	0.52	0.27	1.04	73.77
Absence costs	0.02	0.01	0.03	71.75
Travel costs	1.08	0.50	2.38	78.77
Total cost of dengue per participant	6.69	3.26	14.26	77.15

Data shown as cost per participant using 2014 US$ unless otherwise stated.

APPENDIX 4: THAILAND

Supplemental Table 10

Unit costs and source data (2014 US$)

Cost type	Unit cost (US$)	Source
Thailand		
Hospitalization cost bed per day public	132.86	Suaya et al.[9]; adjusted 2014 US$
Outpatient consultation public	19.93	Suaya et al.[9]; adjusted 2014 US$
Ambulatory Consultation public	19.93	Suaya et al.[9]; adjusted 2014 US$
Daily wage	12.27	World Bank GNIPC[11]
Personal car (hypothesis travel 10 km)	1.02	WHO-CHOICE[13]
Motorcycle (hypothesis travel 10 km)	0.38	WHO-CHOICE[13]
Taxi (cost per travel, hypothesis 10 km in 1/2 hours)	1.64	= Cost per car for 10 km + 1/2 hours of daily wage (WHO-CHOICE[13]; World Bank GNIPC[11])
Public transportation (cost per travel)	0.32	Vietnam cost study Sanofi Pasteur 2012 (Luong et al., 2012); adjusted by 2014 I$ ratio (IMF[4])
Ambulance (cost per travel, hypothesis 10 km in 1/2 hours)	1.64	= Cost per car for 10 km + 1/2 hours of daily wage (WHO-CHOICE[13]; World Bank GNIPC[11])

Supplemental Table 11

Medical and nonmedical resource use and missed school days/workdays attributed to virologically confirmed dengue episodes for all participants (2–14 years) in Thailand

Thailand	All episodes (average)	CYD-TDV group (average)	Control group (average)	*P* value *t* test
Hospitalized	26 (28.6%)	13 (28.9%)	13 (27.7%)	
Length of stay (hospitalization days)	2.8	3.0	2.7	NS
Consultations (*N*)	4.1	4.3	3.9	NS
Workdays lost	3.3	3.9	2.8	NS
School days missed	3.8	3.8	3.7	NS
Nonhospitalized	65 (71.4%)	31 (71.1%)	34 (72.3%)	
Consultations (*N*)	3.0	2.9	3.1	NS
Workdays lost	1.9	1.8	2.1	NS
School days missed	2.6	2.5	2.6	NS
All cases	91	44	47	
Length of stay (hospitalization days)	0.8	0.9	0.7	NS
Consultations (*N*)	3.3	3.3	3.3	NS
Workdays lost	2.3	2.4	2.3	NS
School days missed	2.9	2.9	2.9	NS

NS = not significant.

Supplemental Table 12

Costs associated with virologically confirmed dengue episodes (hospitalized and nonhospitalized combined) for all participants (2–14 years) in Thailand

	All participants (average)	CYD-TDV group (average)	Control group (average)	Reduction (%)
Thailand				
Incidence (%)	6.07	3.57	11.35	
Hospitalization costs	7.00	4.21	12.84	67.23
Consultation costs	4.56	2.62	8.71	69.92
Outpatient consultations	4.05	2.35	7.70	69.54
Ambulatory consultations	0.50	0.27	1.01	72.79
Absence costs	0.08	0.04	0.16	73.24
Travel costs	3.89	2.31	7.23	68.05
Total cost of dengue per participant	15.53	9.18	28.94	68.28

Data shown as cost per participant using 2014 US$ unless otherwise stated.

APPENDIX 5: VIETNAM

Supplemental Table 13

Unit costs and source data (2014 US$)

Cost type	Unit cost (US$)	Source
Vietnam		
Hospitalization cost bed per day public	13.36	Vietnam cost study Sanofi Pasteur 2012 (Luong et al., 2012); adjusted 2014 US$
Outpatient consultation public	4.21	Vietnam cost study Sanofi Pasteur 2012 (Luong et al., 2012); adjusted 2014 US$
Ambulatory consultation public	4.21	Vietnam cost study Sanofi Pasteur 2012 (Luong et al., 2012); adjusted 2014 US$
Daily wage	3.83	World Bank GNIPC[11]
Personal car (hypothesis travel 10 km)	0.92	WHO-CHOICE[13]
Motorcycle (hypothesis travel 10 km)	0.32	WHO-CHOICE[13]
Taxi (cost per travel, hypothesis 10 km in 1/2 hours)	1.11	= Cost per car for 10 km + 1/2 hours of daily wage (WHO-CHOICE[13]; World Bank GNIPC[11])
Public transportation (cost per travel)	0.08	Vietnam cost study Sanofi Pasteur 2012 (Luong et al., 2012)
Ambulance (cost per travel, hypothesis 10 km in 1/2 hours)	1.11	= Cost per car for 10 km + 1/2 hours of daily wage (WHO-CHOICE[13]; World Bank GNIPC[11])

Supplemental Table 14

Medical and nonmedical resource use and missed school days/workdays attributed to virologically confirmed dengue episodes for all participants (2–14 years) in Vietnam

Vietnam	All episodes (average)	CYD-TDV group (average)	Control group (average)	*P* value *t* test
Hospitalized	7 (6.8%)	4 (7.7%)	3 (5.9%)	
Length of stay (hospitalization days)	3.9	4.0	3.7	NS
Consultations (*N*)	3.6	3.5	5.5	NS
Workdays lost	2.9	1.5	4.7	NS
School days missed	3.9	2.5	5.7	NS
Nonhospitalized	96 (93.2%)	48 (92.3%)	48 (94.1%)	
Consultations (*N*)	2.4	2.4	2.4	NS
Workdays lost	0.6	0.6	0.5	NS
School days missed	0.7	0.8	0.6	NS
All cases	103	52	51	
Length of stay (hospitalization days)	0.3	0.3	0.2	NS
Consultations (*N*)	2.5	2.5	2.6	NS
Workdays lost	0.8	0.7	0.7	NS
School days missed	0.9	0.9	0.9	NS

NS = not significant.

Supplemental Table 15

Costs associated with virologically confirmed dengue episodes (hospitalized and nonhospitalized combined) for all participants (2–14 years) in Vietnam

	All participants (average)	CYD-TDV group (average)	Control group (average)	Reduction (%)
Vietnam				
Incidence (%)	4.53	2.83	7.82	
Hospitalization costs	0.16	0.12	0.23	48.39
Consultation costs	0.51	0.32	0.86	62.38
Outpatient consultations	0.47	0.29	0.81	63.95
Ambulatory consultations	0.04	0.03	0.05	37.91
Absence costs	0.01	0.01	0.01	60.44
Travel costs	0.29	0.18	0.50	64.52
Total cost of dengue per participant	0.96	0.62	1.60	61.07

Data shown as cost per participant using 2014 US$ unless otherwise stated.

APPENDIX 6: BRAZIL

Supplemental Table 16

Unit costs and source data (2014 US$)

Cost type	Unit cost (US$)	Source
Brazil		
Hospitalization cost bed per day public	77.67	Martelli et al.[6]/Average number of hospitalization days Martelli et al.[6]; adjusted 2014 US$
Outpatient consultation public	15.73	Martelli et al.[6]/Average number of consultations Martelli et al, 2015; adjusted 2014 US$
Ambulatory consultation public	15.73	Martelli et al.[6]/Average number of consultations Martelli et al, 2015; adjusted 2014 US$
Daily wage	31.49	World Bank GNIPC[12] adjusted 2014 US$
Personal car (hypothesis travel 10 km)	1.37	WHO-CHOICE Unit price 2005
Motorcycle (hypothesis travel 10 km)	0.50	WHO-CHOICE Unit price 2005
Taxi (cost per travel, hypothesis 10 km in 1/2 hours)	2.94	= Cost per car for 10 km + 1/2 hours of daily wage (WHO-CHOICE Unit price 2005; World Bank GNIPC[12])
Public transportation (cost per travel)	0.08	= Average public transport fare Colombia, adjusted by 2014 I$ ratio (IMF[4])
Ambulance (cost per travel, hypothesis 10 km in 1/2 hours)	2.94	= Cost per car for 10 km + 1/2 hours of daily wage (WHO-CHOICE Unit price 2005; World Bank GNIPC[12])

Supplemental Table 17

Medical and nonmedical resource use and missed school days/workdays attributed to virologically confirmed dengue episodes for all participants (9–16 years) in Brazil

Brazil	All episodes (average)	CYD-TDV group (average)	Control group (average)	*P* value *t* test
Hospitalized	5 (4.2%)	1 (2.6%)	4 (4.9%)	
Length of stay (hospitalization days)	2.2	1.0	2.5	NS
Consultations (*N*)	2.4	2.0	2.5	NS
Workdays lost	3	3.0	3.0	NS
School days missed	4	3.0	4.3	NS
Nonhospitalized	114 (95.8%)	37 (97.4%)	77 (95.1%)	
Consultations (*N*)	2.3	2.3	2.3	NS
Workdays lost	0.3	0.5	0.3	NS
School days missed	3.2	2.5	3.7	NS
All cases	119	38	81	
Length of stay (hospitalization days)	0.1	0.0	0.1	NS
Consultations (*N*)	2.3	2.3	2.3	NS
Workdays lost	0.5	0.6	0.4	NS
School days missed	3.3	2.5	3.7	NS

NS = not significant.

Supplemental Table 18

Costs associated with virologically confirmed dengue episodes (hospitalized and nonhospitalized combined) for all participants (9–16 years) in Brazil

	All participants (average)	CYD-TDV group (average)	Control group (average)	Reduction (%)
Brazil				
Incidence (%)	3.35	1.60	6.88	
Hospitalization costs	0.24	0.03	0.66	95.03
Consultation costs	1.22	0.57	2.52	77.38
Outpatient consultations	1.05	0.48	2.18	77.74
Ambulatory consultations	0.17	0.09	0.35	75.15
Absence costs	0.07	0.04	0.12	67.36
Travel costs	3.12	1.40	6.58	78.78
Total cost of dengue per participant	4.64	2.04	9.88	79.37

Data shown as cost per participant using 2014 US$ unless otherwise stated.

APPENDIX 7: COLOMBIA

Supplemental Table 19

Unit costs and source data (2014 US$)

Cost type	Unit cost (US$)	Source
Colombia		
Hospitalization cost bed per day public	85.41	= (Castro Rodriguez et al.[1]/Average hospitalization cost per case/Average number of hospitalization days CYD15 trial); adjusted 2014 US$
Outpatient consultation public	25.38	= (Castro Rodriguez et al.[1]/Average ambulatory cost per case/Average number of consultation CYD15 trial); adjusted 2014 US$
Ambulatory consultation public	25.38	= (Castro Rodriguez et al.[1]/Average ambulatory Cost per case/Average number of consultation CYD15 trial); adjusted 2014 US$
Daily wage	19.12	World Bank GNIPC[12] adjusted 2014 US$
Personal car (hypothesis travel 10 km)	1.19	WHO-CHOICE Unit price 2005
Motorcycle (hypothesis travel 10 km)	0.43	WHO-CHOICE Unit price 2005
Taxi (cost per travel, hypothesis 10 km in 1/2 hours)	2.15	= Cost per car for 10 km + 1/2 hours of daily wage (WHO-CHOICE Unit price 2005; World Bank GNIPC[12])
Public transportation (cost per travel)	0.30	= Average public transport fare Colombia
Ambulance (cost per travel, hypothesis 10 km in 1/2 hours)	2.15	= Cost per car for 10 km + 1/2 hours of daily wage (WHO-CHOICE Unit price 2005; World Bank GNIPC[12])

Supplemental Table 20

Medical and nonmedical resource use and missed school days/workdays attributed to virologically confirmed dengue episodes for all participants (9–16 years) in Colombia

Colombia	All episodes (average)	CYD-TDV group (average)	Control group (average)	*P* value *t* test
Hospitalized	35 (12.7%)	7 (6.3%)	28 (17%)	
Length of stay (hospitalization days)	3.8	4.7	3.5	NS
Consultations (*N*)	2.8	3.0	2.8	NS
Workdays lost	2.9	2.0	3.1	NS
School days missed	6.8	7.0	6.7	NS
Nonhospitalized	241 (87.3%)	104 (93.7%)	137 (83%)	
Consultations (*N*)	2.6	2.6	2.6	NS
Workdays lost	0.4	0.3	0.4	NS
School days missed	2.8	2.7	2.9	NS
All cases	276	111	165	
Length of stay (hospitalization days)	0.5	0.3	0.6	NS
Consultations (*N*)	2.6	2.6	2.6	NS
Workdays lost	0.7	0.4	0.9	< 0.01
School days missed	3.3	2.9	3.5	NS

NS = not significant.

Supplemental Table 21

Costs associated with virologically confirmed dengue episodes (hospitalized and nonhospitalized combined) for all participants (9–16 years) in Colombia

	All participants (average)	CYD-TDV group (average)	Control group (average)	Reduction (%)
Colombia				
Incidence (%)	2.83	1.71	5.08	
Hospitalization costs	1.16	0.43	2.61	83.35
Consultation costs	1.89	1.13	3.42	66.96
Outpatient consultations	1.56	0.93	2.82	66.92
Ambulatory consultations	0.33	0.20	0.59	67.13
Absence costs	0.10	0.06	0.19	68.20
Travel costs	1.64	0.81	3.32	75.73
Total cost of dengue per participant	4.80	2.43	9.54	74.52

Data shown as cost per participant using 2014 US$ unless otherwise stated.

APPENDIX 8: HONDURAS

Supplemental Table 22

Unit costs and source data (2014 US$)

Cost type	Unit cost (US$)	Source
Honduras		
Hospitalization cost bed per day public	58.20	= (Shepard et al.[7]/Average hospitalization cost per case/Average number of hospitalization days CYD15 trial); adjusted 2014 US$
Outpatient consultation public	15.77	= (Shepard et al.[7]/Average consultation cost per case/Average number of consultation CYD15 trial); adjusted 2014 US$
Ambulatory consultation public	15.77	= (Shepard et al.[7]/Average consultation cost per case/Average number of consultation CYD15 trial); adjusted 2014 US$
Daily wage	6.20	World Bank GNIPC[12] adjusted 2014 US$
Personal car (hypothesis travel 10 km)	1.20	WHO-CHOICE unit price 2005
Motorcycle (hypothesis travel 10 km)	0.43	WHO-CHOICE unit price 2005
Taxi (cost per travel, hypothesis 10 km in 1/2 hours)	1.51	= Cost per car for 10 km + 1/2 hours of daily wage (WHO-CHOICE Unit price 2005; World Bank GNIPC[12])
Public transportation (cost per travel)	0.118	= Average public transport fare Colombia, adjusted by 2014 I$ ratio (IMF[4])
Ambulance (cost per travel, hypothesis 10 km in 1/2 hours)	1.51	= Cost per car for 10 km + 1/2 hours of daily wage (WHO-CHOICE Unit price 2005; World Bank GNIPC[12])

Supplemental Table 23

Medical and nonmedical resource use and missed school days/workdays attributed to virologically confirmed dengue episodes for all participants (9–16 years) in Honduras

Honduras	All episodes (average)	CYD-TDV group (average)	Control group (average)	*P* value *t* test
Hospitalized	11 (9.6%)	4 (9.5%)	7 (9.6%)	
Length of stay (hospitalization days)	4.1	4.0	4.1	NS
Consultations (*N*)	2.2	2.5	2.0	NS
Workdays lost	0.3	0.8	0.0	NS
School days missed	5.3	6.3	4.7	NS
Nonhospitalized	104 (90.4%)	38 (90.5%)	66 (90.4%)	
Consultations (*N*)	2.5	2.5	2.5	NS
Workdays lost	0.0	0.0	0.1	NS
School days missed	1.7	1.9	1.6	NS
All cases	115	42	73	
Length of stay (hospitalization days)	0.4	0.4	0.4	NS
Consultations (*N*)	2.5	2.5	2.5	NS
Workdays lost	0.1	0.1	0.1	NS
School days missed	2.1	2.3	1.9	NS

NS = not significant.

Supplemental Table 24

Costs associated with virologically confirmed dengue episodes (hospitalized and nonhospitalized combined) for all participants (9–16 years) in Honduras

	All participants (average)	CYD-TDV group (average)	Control group (average)	Reduction (%)
Honduras				
Incidence (%)	4.11	2.25	7.82	
Hospitalization costs	0.79	0.42	1.53	72.41
Consultation costs	2.40	1.31	4.58	71.43
Outpatient consultations	1.84	1.02	3.47	70.65
Ambulatory consultations	0.56	0.29	1.11	73.86
Absence costs	0.07	0.04	0.12	67.74
Travel costs	0.31	0.18	0.58	68.39
Total cost of dengue per participant	3.57	1.95	6.81	71.33

Data shown as cost per participant using 2014 US$ unless otherwise stated.

APPENDIX 9: MEXICO

SUPPLEMENTAL TABLE 25

Unit costs and source data (2014 US$)

Cost type	Unit cost (US$)	Source
Mexico		
Hospitalization cost bed per day public	243.12	= Undurraga et al.[10]; adjusted 2014 US$
Outpatient consultation public	69.24	= Undurraga et al.[10]; adjusted 2014 US$
Ambulatory consultation public	69.24	= Undurraga et al.[10]; adjusted 2014 US$
Daily wage	26.87	World Bank GNIPC[12] adjusted 2014 US$
Personal car (hypothesis travel 10 km)	1.32	WHO-CHOICE unit price 2005
Motorcycle (hypothesis travel 10 km)	0.48	WHO-CHOICE unit price 2005
Taxi (cost per travel, hypothesis 10 km in 1/2 hours)	2.66	= Cost per car for 10 km + 1/2 hours of daily wage (WHO-CHOICE Unit price 2005; World Bank GNIPC[12])
Public transportation (cost per travel)	0.57	= Average public transport fare Colombia, adjusted by 2014 I$ ratio (IMF[4])
Ambulance (cost per travel, hypothesis 10 km in 1/2 hours)	2.66	= Cost per car for 10 km + 1/2 hours of daily wage (WHO-CHOICE Unit price 2005; World Bank GNIPC[12])

SUPPLEMENTAL TABLE 26

Medical and nonmedical resource use and missed school days/workdays attributed to virologically confirmed dengue episodes for all participants (9–16 years) in Mexico

Mexico	All episodes (average)	CYD-TDV group (average)	Control group (average)	*P* value *t* test
Hospitalized	6 (4.4%)	3 (3.8%)	3 (5.3%)	
Length of stay (hospitalization days)	5.2	6.7	3.7	< 0.05
Consultations (*N*)	1.3	2.0	0.7	NS
Workdays lost	1.8	3.7	0.0	NS
School days missed	2.5	3.3	1.7	NS
Nonhospitalized	130 (95.6%)	76 (96.2%)	54 (94.7%)	
Consultations (*N*)	2.1	2.1	2.1	NS
Workdays lost	0.2	0.2	0.3	NS
School days missed	1.5	1.3	1.9	NS
All cases	136	79	57	
Length of stay (hospitalization days)	0.2	0.3	0.2	NS
Consultations (*N*)	2.1	2.1	2.1	NS
Workdays lost	0.3	0.3	0.3	NS
School days missed	1.5	1.3	1.8	NS

NS = not significant.

SUPPLEMENTAL TABLE 27

Costs associated with virologically confirmed dengue episodes (hospitalized and nonhospitalized combined) for all participants (9–16 years) in Mexico

	All participants (average)	CYD-TDV group (average)	Control group (average)	Reduction (%)
Mexico				
Incidence (%)	3.93	3.42	4.95	
Hospitalization costs	2.18	2.10	2.32	9.41
Consultation costs	5.62	4.88	7.09	31.17
Outpatient consultations	5.46	4.70	6.97	32.56
Ambulatory consultations	0.16	0.18	0.12	−49.48
Absence costs	0.08	0.07	0.10	32.20
Travel costs	1.64	1.35	2.22	39.16
Total cost of dengue per participant	9.51	8.40	11.73	28.38

Data shown as cost per participant using 2014 US$ unless otherwise stated.

APPENDIX 10: PUERTO RICO

Supplemental Table 28

Unit costs and source data (2014 US$)

Cost type	Unit cost (US$)	Source
Puerto Rico		
Hospitalization cost bed per day public	849.82	= (Halasa et al.[3]/Average hospitalization cost per case/Average number of hospitalization days); adjusted 2014 US$
Outpatient consultation public	194.75	= (Halasa et al.[3]/Average consultation Cost per case/Average number of consultation CYD15 trial); adjusted 2014 US$
Ambulatory consultation public	194.75	= (Halasa et al.[3]/Average consultation cost per case/Average number of consultation CYD15 trial); adjusted 2014 US$
Daily wage	53.87	World Bank GNIPC[12]; adjusted 2014 US$
Personal car (hypothesis travel 10 km)	1.52	WHO-CHOICE unit price 2005
Motorcycle (hypothesis travel 10 km)	0.75	WHO-CHOICE unit price 2005
Taxi (cost per travel, hypothesis 10 km in 1/2 hours)	4.21	= Cost per car for 10 km + 1/2 hours of daily wage (WHO-CHOICE Unit price 2005; World Bank GNIPC[12])
Public transportation (cost per travel)	2.13	= Average public transport fare Colombia, adjusted by 2014 I$ ratio (IMF[4])
Ambulance (cost per travel, hypothesis 10 km in 1/2 hours)	4.21	= Cost per car for 10 km + 1/2 hours of daily wage (WHO-CHOICE Unit price 2005; World Bank GNIPC[12])

Supplemental Table 29

Medical and nonmedical resource use and missed school days/workdays attributed to virologically confirmed dengue episodes for all participants (9–16 years) in Puerto Rico

Puerto-Rico	All episodes (average)	CYD-TDV group (average)	Control group (average)	*P* value *t* test
Hospitalized	3 (12.5%)	2 (18.2%)	1 (7.7%)	
Length of stay (hospitalization days)	5.3	4	8	NS
Consultations (*N*)	2.0	2	2	NS
Workdays lost	0.0	0	0	NS
School days missed	7.3	7.5	7	NS
Nonhospitalized	21 (87.5%)	9 (81.8%)	12 (92.3%)	
Consultations (*N*)	2.0	2	2	NS
Workdays lost	0.1	0	0.2	NS
School days missed	2.1	2	2.2	NS
All cases	24	11	13	
Length of stay (hospitalization days)	0.7	0.7	0.6	NS
Consultations (*N*)	2.0	2.0	2.0	NS
Workdays lost	0.1	0.0	0.2	NS
School days missed	2.7	3.0	2.5	NS

NS = not significant.

Supplemental Table 30

Costs associated with virologically confirmed dengue episodes (hospitalized and nonhospitalized combined) for all participants (9–16 years) in Puerto Rico

	All participants (average)	CYD-TDV group (average)	Control group (average)	Reduction (%)
Puerto Rico				
Incidence (%)	1.83	1.26	2.95	
Hospitalization costs	10.34	7.77	15.45	49.71
Consultation costs	7.11	4.90	11.51	57.45
Outpatient consultations	6.96	4.90	11.07	55.75
Ambulatory consultations	0.15	0.00	0.44	100.00
Absence costs	0.06	0.04	0.09	57.45
Travel costs	1.93	1.54	2.69	42.86
Total cost of dengue per participant	19.43	14.24	29.74	52.11

Data shown as cost per participant using 2014 US$ unless otherwise stated.

APPENDIX 11: ALL COUNTRIES ASSESSED

Supplemental Table 31

Medical and nonmedical resource use and missed school days/workdays attributed to virologically confirmed dengue (all ages and countries combined)

	All participants (average)	CYD-TDV group (average)	Control group (average)	*P* value *t* test
Hospitalized	158 (12.4%)	104 (14.7%)	54 (9.5%)	0.004
Length of stay (hospitalization days)	4.1	4.0	4.0	NS
Consultations (*N*)	3.2	2.8	3.3	0.2
Workdays lost	2.1	2.2	2.0	ns
School days missed	4.9	5.1	4.4	0.44
Nonhospitalized	1121 (87.6%)	604 (85.3%)	517 (90.5%)	
Consultations (*N*)	2.4	2.4	2.3	0.1
Workdays lost	0.4	0.3	0.3	NS
School days missed	1.7	1.8	1.6	0.1
All episodes	1279	708	571	
Length of stay (hospitalization days)	0.5	0.6	0.4	
Consultations (*N*)	2.5	2.5	2.4	
Workdays lost	0.6	0.6	0.5	
School days missed	2.1	2.3	1.8	0.004*

NS = not significant

Supplemental Table 32

Average unit costs in I$ 2014 used for the cost analysis in CYD14 and CYD15

Cost type	Brazil	Colombia	Honduras	Mexico	Puerto	Indonesia	Malaysia	Philippines	Thailand	Vietnam	Country average
Hospitalization cost bed per day public	125.65	169.43	201.88	371.62	849.82	114.88	509.75	140.89	229.93	53.32	276.72
Outpatient consultation public	25.45	50.26	54.70	105.84	194.75	27.84	97.60	45.08	34.49	16.78	65.28
Ambulatory consultation	25.45	50.26	54.70	105.84	194.75	27.84	97.60	45.08	34.49	16.78	65.28
Daily wage	50.94	37.85	21.49	41.08	53.87	13.74	45.06	13.22	21.24	15.28	31.38
Personal car (hypothesis travel 10 km)	2.22	2.37	4.15	2.01	1.52	1.89	2.26	2.51	1.77	3.67	2.44
Motorcycle (hypothesis travel 10 km)	0.80	0.86	1.50	0.73	0.75	0.34	0.78	0.87	0.65	1.27	0.86
Taxi (cost per travel, hypothesis 10 km in 1/2 hours)	4.76	4.26	5.22	4.06	4.21	2.58	4.51	3.17	2.83	4.43	4.00
Public transportation (cost per travel)	0.12	0.59	0.41	0.87	2.13	0.27	1.25	0.25	0.56	0.33	0.68
Ambulance (cost per travel, hypothesis 10 km in 1/2 hours)	4.76	4.26	5.22	4.06	4.21	2.58	4.51	3.17	2.83	4.43	4.00

APPENDIX 12: CALCULATION OF INTERNATIONAL DOLLAR (I$)

Supplemental Table 33

Relationship between International dollars (I$) and U.S. dollars (US$)

Public	PPP rate 2014 IMF 1 I$ = *x* LCU	2014 IMF LCU = *x* US$	US$ to I$
Brazil	0.395	0.639000742	1.6177234
Colombia	417.434	826.5611193	1.98010013
Honduras	2.424	8.408302808	3.46877179
Mexico	2.208	3.375114705	1.52858456
Puerto	1	1	1
Indonesia	1,054.949	2160.796814	2.04824765
Malaysia	1.43	2.624315531	1.83518569
Philippines	13.36	26.41738968	1.97734953
Thailand	14.532	25.14996397	1.73066088
Vietnam	2,748.833	10,967.13557	3.9897424

PPP = purchasing power parity. Based on International Monetary Fund (IMF) statistics, we converted I$ into the local current unit (LCU; year 2014 reference) and then converted them in US$. This allowed us to have US$ converted into I$. Source: IMF[4].

APPENDIX 13: RESOURCE USE AND ASSOCIATED COSTS IN PARTICIPANTS AGED < 9 YEARS

Supplemental Table 34

Summary of resource use comparison between participants with virologically confirmed dengue episodes in the two study groups (participants aged < 9 years, all countries combined)

	All groups < 9 years (average)	CYD-TDV (average)	Control group (average)	*P* value *t* test
Hospitalized	62 (16.4%)	28	34	
Length of stay (hospitalization days)	4.3	3.6	4.8	0.030
Consultations (*N*)	3.5	3.4	3.6	0.049
Workdays lost	1.9	1.9	1.9	0.403
School days missed	4.9	4.9	4.9	0.019
Nonhospitalized	316 (83.6%)	170	146	
Consultations (*N*)	2.4	2.4	2.5	
Workdays lost	0.6	0.5	0.6	
School days missed	1.7	1.5	2.0	
All episodes*	378	198	180	
Length of stay (hospitalization days)	0.7	0.5	0.9	
Consultations (*N*)	2.6	2.6	2.7	
Workdays lost	0.8	0.7	0.9	
School days missed	2.3	2.0	2.6	

* Weighted average.

Supplemental Table 35

Costs associated with virologically confirmed dengue episodes (participants aged < 9 years, all countries combined)

	< 9 years all groups (average)	CYD-TDV group (average)	Control group (average)	*P* value *t* test
Hospitalized episodes (all countries combined)				
Hospitalization costs	1,182.75	1,008.05	1,326.62	
Consultation costs	273.84	267,17	279.33	
Outpatient consultations	231.63	226,14	236.15	
Ambulatory consultations	42.21	41,03	43.17	
Travel costs	3.55	2,90	4.08	
Absence costs	212.55	214,04	211.33	
Total cost per dengue episode	1,672.69	1,492.16	1,821.37	
Nonhospitalized episodes (all countries combined)				
Consultation costs	175.43	174.34	176.70	
Outpatient consultations	159.27	157.82	160.96	
Ambulatory consultations	16.16	16.52	15.74	
Travel costs	1.47	1.32	1.66	
Absence costs	72.68	63.49	83.39	
Total cost per dengue episode	249.58	239.15	261.74	
All episodes*				
Hospitalization costs	194.00	142.55	250.58	0.031
Consultation costs	191.57	187.47	196.08	0.113
Outpatient consultations	171.14	167.48	175.16	
Ambulatory consultations	20.43	19.98	20.92	
Travel costs	1.81	1.54	2.11	0.006
Absence costs	95.63	84.78	107.55	0.039
Total cost per dengue episode	483.00	416.34	556.33	0.014

* Weighted average.
Data shown as cost per dengue episode using 2014 I$.

SUPPLEMENTAL REFERENCES

1. Castro Rodriguez R, Galera-Gelvez K, Lopez Yescas JG, Rueda-Gallardo JA, 2015. Costs of dengue to the health system and individuals in Colombia from 2010 to 2012. *Am J Trop Med Hyg 92:* 709–714.
2. Edillo FE, Halasa YA, Largo FM, Erasmo JN, Amoin NB, Alera MT, Yoon IK, Alcantara AC, Shepard DS, 2015. Economic cost and burden of dengue in the Philippines. *Am J Trop Med Hyg 92:* 360–366.
3. Halasa YA, Shepard DS, Zeng W, 2012. Economic cost of dengue in Puerto Rico. *Am J Trop Med Hyg 86:* 745–752.
4. International Monetary Fund (IMF), 2014. *World Economic and Financial Surveys; World Economic Outlook Database*. Available at: https://www.imf.org/external/pubs/ft/weo/2014/01/weodata/index.aspx. Accessed June 14, 2014.
5. Luong QC et al., 2012. *Assessing the Economic Burden of Dengue in Southern Vietnam: Results of a Prospective Multicenter Cost Study*. Ho Chi Minh City, Vietnam: Pasteur Institute.
6. Martelli CM et al., 2015. Economic impact of dengue: multicenter study across four Brazilian regions. *PLoS Negl Trop Dis 9:* e0004042.
7. Shepard DS, Undurraga EA, Halasa YA, Stanaway JD, 2016. The global economic burden of dengue: a systematic analysis. *Lancet Infect Dis 16:* 935–941.
8. Shepard DS, Undurraga EA, Lees RS, Halasa Y, Lum LC, Ng CW, 2012. Use of multiple data sources to estimate the economic cost of dengue illness in Malaysia. *Am J Trop Med Hyg 87:* 796–805.
9. Suaya JA et al., 2009. Cost of dengue cases in eight countries in the Americas and Asia: a prospective study. *Am J Trop Med Hyg 80:* 846–855.
10. Undurraga EA et al., 2015. Economic and disease burden of dengue in Mexico. *PLoS Negl Trop Dis 9:* e0003547.
11. World Bank GNIPC (Gross national income per capita 2010, Atlas method and PPP), 2010. *World Development Indicators Database*. Available at: http://siteresources.worldbank.org/DATASTATISTICS/Resources/GNIPC.pdf. Accessed July 1, 2011.
12. World Bank GNIPC (Gross national income per capita 2013, Atlas method and PPP), 2013. *World Development Indicators Database.* Available at: http://www.gavi.org/library/gavi-documents/guidelines-and-forms/gni-per-capita-2013/. Accessed November 6, 2014.
13. World Health Organization, 2011. *Cost Effectiveness and Strategic Planning (WHO-CHOICE). Quantities and Unit Prices (Cost Inputs)*. Available at: http://www.who.int/choice/cost-effectiveness/inputs/en/. Accessed April 30, 2014.
